# A computational roadmap to activity-dependent myelination

**DOI:** 10.3389/fncom.2026.1895171

**Published:** 2026-09-25

**Authors:** Thomas Nguyen, Jeremie Lefebvre

**Affiliations:** 1 Department of Biology, University of Ottawa, Ottawa, ON, Canada; 2 Krembil Brain Institute, University Health Network, Toronto, ON, Canada; 3 Department of Physics, University of Ottawa, Ottawa, ON, Canada; 4 Department of Mathematics, University of Toronto, Toronto, ON, Canada

**Keywords:** activity-dependent myelination, conduction delays, myelin, neural computation, neural timing, oligodendrocytes

## Abstract

There is growing evidence to support myelin plasticity as a complementary mechanism to synaptic plasticity in the brain. Activity-dependent myelination (ADM) enables oligodendrocytes to modify myelin structure in response to neural activity, modulating axonal conduction velocity and delays to maintain precise signal timing. This introduces a timing-based plasticity mechanism that operates in parallel to changes in signal strength. Despite its significance, ADM lacks a strong theoretical framework due to the interaction of multiple mechanisms operating on widely different time scales. Computational models are beginning to address this gap by providing powerful tools to interpret and guide experimental studies. This perspective explores the growing number of computational ADM models, comparing how they link neural activity to myelin adaptation, and their assumptions, strengths, and limitations. We also discuss the challenges of modeling ADM and open questions, positioning ADM as a key component of neural plasticity.

## 1 Introduction

Research on brain plasticity and learning has historically focused on the synapse, resulting in the discovery of mechanisms such as spike-timing-dependent plasticity (Dan and Poo, 2006), long- and short-term synaptic potentiation and depression (Paulsen and Sejnowski, 2000), and Hebbian learning (Hebb and Hebb, 1961; Citri and Malenka, 2008). These discoveries and their associated models have substantially advanced our understanding of how neural activity modifies synaptic strength and have led to insightful computational frameworks that formalize synaptic plasticity, driving major progress in neuroscience and inspiring engineering applications such as machine learning. However, efficient communication in the brain, as in other complex systems, is also critically dependent on precise timing. Temporal coordination of neural signaling is largely supported by myelin, a lipid-rich structure that optimizes the speed and efficiency of action potential propagation along axons by enabling saltatory conduction (Stadelmann et al., 2019).

Myelin sheath formation in the brain is carried out by specialized glial cells known as oligodendrocytes (OLs) (Stadelmann et al., 2019). This process is most active during development, when oligodendrocyte precursor cells (OPCs) migrate throughout the central nervous system and then mature into functional OLs, each forming several myelin sheaths around multiple axons (Simons and Nave, 2016). Myelination is characterized by a period of dynamic sheath formation and loss, followed by a pruning period (Czopka et al., 2013; Liu et al., 2013). The formation of myelin during these periods has been shown to be influenced by neural activity. Specifically, increased neural activity appears to enhance OPC proliferation and maturation, subsequently increasing myelination (Wake et al., 2011; Gibson et al., 2014; Braaker et al., 2025). Following these developmental stages, myelin structure was classically thought to be highly stable (Czopka et al., 2013), with changes in myelin structure occurring primarily in response to the development of new OLs or myelin-affecting pathologies.

However, more recent experimental evidence challenges this view, revealing that myelination remains dynamic throughout adulthood (Fields, 2005; Young et al., 2013; Pan et al., 2020; Xin and Chan, 2020; Osso and Hughes, 2024). Indeed, increasing experimental (Gibson et al., 2014; Hughes et al., 2018; Auer et al., 2018; Mitew et al., 2018; Bacmeister et al., 2020, 2022) and computational (Pajevic et al., 2014; Noori et al., 2020; Talidou et al., 2022; Pajevic et al., 2023) evidence suggests that neural activity influences OL activity, and vice versa, forming a neuron-glia feedback loop that shapes the myelination program of the CNS in real time. This understudied phenomenon, termed *activity-dependent myelination* (ADM), is a form of brain plasticity that bidirectionally and reversibly modifies myelin structure in response to neural activity to coordinate the timing of neural signaling and support the metabolic needs of the axon (Fields, 2015; Knowles et al., 2022a). Although ADM experiments have primarily investigated how increased neural activity promotes increased myelination, there is also growing evidence for activity-dependent myelin retraction (Makinodan et al., 2012; Dutta et al., 2018; Bacmeister et al., 2022). Studies in the rodent cortex reveal that learning, experience, and behavior alter myelin sheath properties such as length (Auer et al., 2018; Bacmeister et al., 2022), number of sheaths (Gibson et al., 2014; Hughes et al., 2018; Mitew et al., 2018; Bacmeister et al., 2020, 2022), thickness (Mitew et al., 2018), and organization (Hughes et al., 2018; Auer et al., 2018; Mitew et al., 2018; Bacmeister et al., 2020, 2022). Complementary computational studies demonstrate that changes in myelination, commonly operationalized through changes in conduction velocity or time delays, optimize action potential arrival timing at postsynaptic targets, thereby modulating spike integration and network-level synchrony (Pajevic et al., 2014; Noori et al., 2020; Talidou et al., 2022; Pajevic et al., 2023).

In the healthy brain, ADM-modulated plasticity optimizes and coordinates neural communication (Jamann et al., 2025), which is essential to support key functions such as learning (Hughes et al., 2018; Bacmeister et al., 2020; Kato et al., 2020; Bacmeister et al., 2022; Kato et al., 2023), memory (Steadman et al., 2020; Xin and Chan, 2020; Pan et al., 2020), and sensory and motor function (Gibson et al., 2014). Abnormal neural activity can disrupt healthy ADM by promoting maladaptive myelination that reinforces these activity patterns, creating a feedback loop of dysfunction linked to neurological disorders (Knowles et al., 2022a). For example, in certain forms of epilepsy, seizure activity has been shown to promote maladaptive hypermyelination, reinforcing seizure activity in the brain and consequently driving disease progression (Knowles et al., 2022b). In demyelinating disorders such as multiple sclerosis, the disruptions caused by compromised myelination underlie the sensory, motor, and cognitive deficits typically associated with the condition (Lubetzki and Stankoff, 2014; Madsen et al., 2022).

In contrast to synaptic plasticity, ADM does not possess a comparably rich history of models and computational frameworks. Despite the growing confluence of evidence supporting its significance, ADM still lacks strong theoretical foundations, limiting experimental interpretation and progress. Like synaptic plasticity (Markram et al., 1997; Bi and Poo, 1998; Sjöström et al., 2001; Song et al., 2000; Feldman, 2012), ADM is governed by a wide range of interacting biological mechanisms, including OL migration and differentiation, developmental stage and mature neuron-glia coupling, molecular signaling, ion channel dynamics, and genetic factors. Collectively, these processes span multiple temporal and spatial scales in the brain. Action potential propagation occurs on the order of milliseconds, whereas changes in myelination are far more gradual, often occurring over hours to days (Czopka et al., 2013). Likewise, changes involved in ADM-related processes range from the subcellular scale to the axon (where neuron-glia interactions occur), to gray matter microcircuits, and to large-scale white matter projecting networks. The significant challenges surrounding ADM highlight the need for interdisciplinary approaches. Computational models are powerful tools for bridging these gaps and have long provided insight into neural dynamics across a variety of systems. They enable rapid testing of predictions informed by experimental data and allow precise manipulation of neuronal parameters at both the single-cell and network levels, conditions that are often difficult to perform experimentally.

Our current theoretical understanding of ADM and its impact on the timing of neural signaling is implicit, rooted in decades of theoretical work on time-delayed neural systems (Choi et al., 2000; Atay, 2003; Frank et al., 2003; Dhamala et al., 2004; Campbell, 2007; Coombes and Laing, 2009; Campbell and Kobelevskiy, 2012; Hutt et al., 2012; Lefebvre and Hutt, 2013; Lefebvre et al., 2025), alongside a vast repertoire of studies exploring how the span and organization of such delays shape the dynamics of neural networks across spatial scales (Meyer et al., 2008; Deco et al., 2009; Cabral et al., 2011, 2014; Sayli and Coombes, 2024). Explicit models of ADM, in which myelination is plastic, remain relatively scarce. Nevertheless, there are some recent studies (Ford et al., 2015; Arancibia-Càrcamo et al., 2017; Pajevic et al., 2014; Noori et al., 2020; Park and Lefebvre, 2020; Talidou et al., 2022; Pajevic et al., 2023; Talidou and Lefebvre, 2025) that capture different aspects of the phenomenon, varying greatly in physiological detail, scale, and scope. This diversity results from the challenge of balancing physiological realism with computational efficiency and mathematical tractability. It also highlights efforts in reconciling phenomena occurring at vastly different temporal and spatial scales. Each of these factors has inspired models that make distinct assumptions and often employ distinct modeling strategies (Figure 1). Given that existing models almost exclusively fall into one of the three spatial scale categories (i.e., axonal, microcircuit, or large-scale; see below), there is limited common ground linking theoretical approaches with experimental observations. A careful and systematic discussion of these models, delineating their respective strengths, limitations, and potential, will help establish how they contribute to our overall understanding of ADM and identify the gaps that remain.

**Figure 1:**
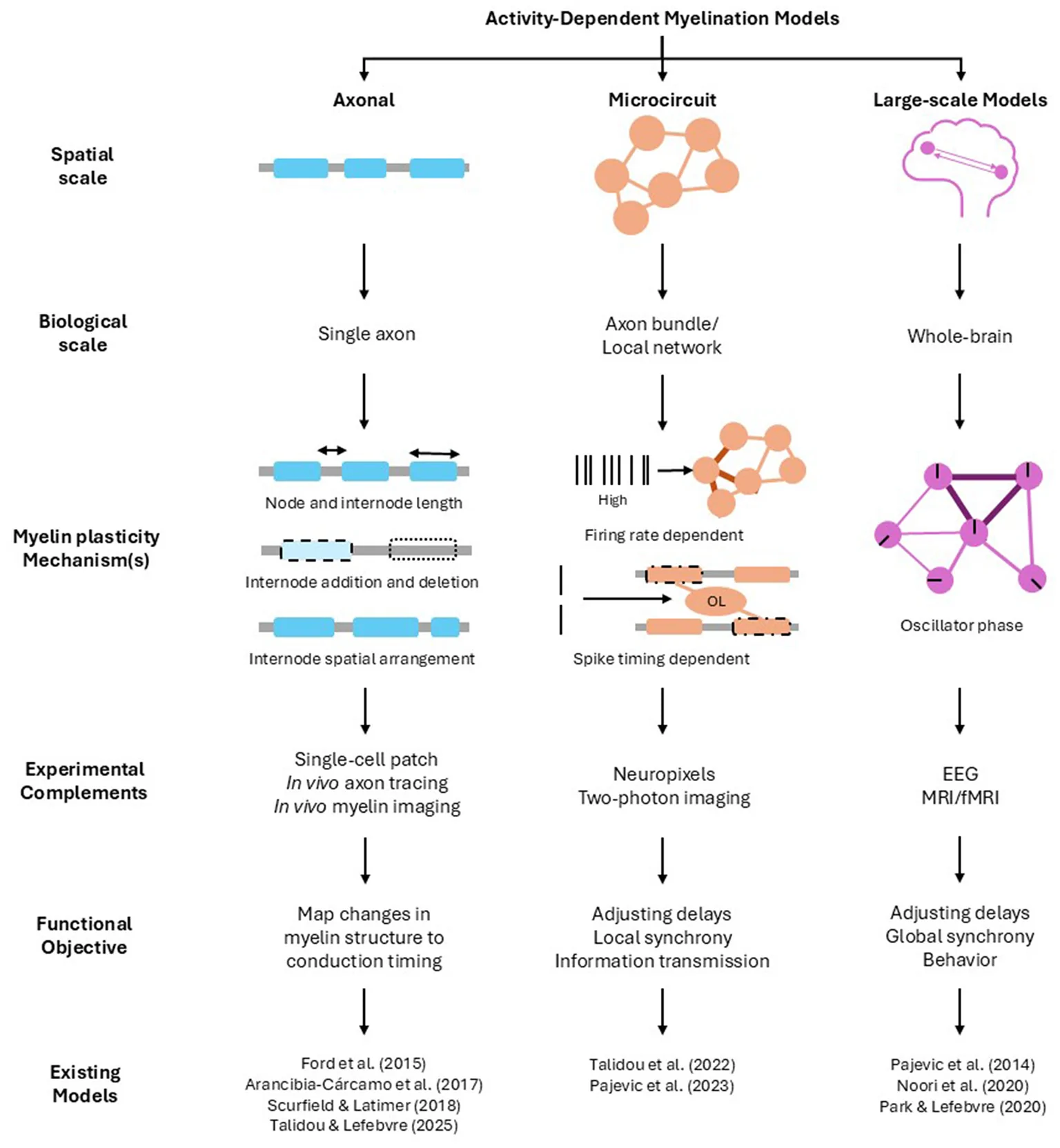
Different implementations of existing activity-dependent myelination models. The three existing classes of models sorted by spatial scale: axonal models (left), microcircuit models (center), and large-scale networks (right). Below details on the plasticity mechanisms, complementary experimental techniques, and objectives of the model type are included. Current models primarily exist along one of the vertical branches, while future modeling should also look to horizontally integrate the different classes of models.

## 2 Computational models of activity-dependent myelination

### 2.1 Axonal models of ADM

The first class of models focuses on individual axons, where neuron-glia interactions occur. These models primarily examine on how changes and differences in myelin structure map onto changes in conduction timing. In the context of ADM, many myelin properties may undergo remodeling in response to neural activity, including node length, internode length, sheath thickness, and sheath formation and loss. Given that structural remodeling of myelin *in vivo* often impacts multiple properties simultaneously, computational models can isolate the impact of individual variables, allowing models to predict their effects on conduction timing and determine where causal relationships exist.

Combined experimental-computational work is particularly valuable since experimental constraints can greatly increase a model's biological realism. For example, Ford et al. (2015) fit their model to geometrical measurements and electrophysiological recordings from gerbils. They integrated these measurements into an axon model to investigate how shorter internode lengths found along low-frequency globular bushy cell auditory fibers (termed GBClat) affect action potential conduction compared to longer internode lengths found in high-frequency auditory fibers (GBCmed). Specifically, Ford et al. (2015) leveraged modeling to establish how the ratio between internode length and the internodal axon diameter (*L*/*d* ratio) influences axonal conduction velocity. The model is based on the Halter and Clark model of the myelinated axon (Halter and Clark, 1991), a multi-compartment model used to simulate the electrical activity of a myelinated axon. It is widely used due to its multi-axial nature, which models the intra-axonal, peri-axonal, and extra-axonal potentials independently, unlike in single-cable models, and its ability to integrate anatomical and electrophysiological data. Hodgkin-Huxley-like kinetics were implemented to emulate ion channel activity and leak conductance, establishing a biophysically detailed model. The smaller *L*/*d* ratio observed in GBClat axons, due to their shorter internode lengths, accounts for a 15% increase in conduction velocity compared to GBCmed axons alone, with other factors contributing to a greater overall difference. Further, for both axon types, axon types exhibit an optimal *L*/*d* ratio that maximizes conduction velocity. Internode lengths that are too short result in lower conduction velocity due to the lack of overall myelination, while depolarizations are not efficiently transferred between nodes when internode lengths are too long. While not explicitly simulating ADM, the ability to lengthen and shorten internodes to optimize conduction velocity is insightful, as it presents a candidate mechanism by which neural activity may modulate spike timing through changes in myelin structure.

Similarly, work building on the results of the aforementioned study (Ford et al., 2015) used computational modeling to explore how changes in node length affect axonal conduction velocity (Arancibia-Càrcamo et al., 2017). A double-cable model derived from the Halter and Clark model (Halter and Clark, 1991; Richardson et al., 2000) was adapted by fitting its model parameters to measurements from the optic nerve and cerebral cortex in rats. The optic nerve contains shorter nodes than the cortex and exhibits less variability in node length between axons. Given a constant number of ion channels at each node, increasing node length almost linearly decreases conduction velocity due to increased capacitance and axial resistance. However, given a constant nodal channel density, there is an optimal node length that maximizes conduction velocity, consistent with findings on internode length (Ford et al., 2015). When channel density is constant, reducing node length also reduces the number of ion channels present at each node, resulting in slower conduction velocity. Thus, computational modeling reveals how micrometer-scale changes in node length can increase conduction velocity in the optic nerve by up to 20%, with an even greater increase in the cortex (Arancibia-Càrcamo et al., 2017). Given that mature internode architecture has long been viewed as stable and metabolically expensive to alter, a central advancement of this study over its predecessor is that nodal remodeling presents a more efficient strategy by which ADM may tune conduction velocity.

Finally, axonal models have examined how spatial heterogeneity in myelin coverage impacts neural signaling, injury susceptibility, and recovery (Scurfield and Latimer, 2018; Talidou and Lefebvre, 2025). Since myelin sheaths vary in length and are often non-uniformly arranged along an axon (Chong et al., 2012; Tomassy et al., 2014), an important area of investigation is how changes in this organization influence spike timing. Although patchy myelination is present across many areas of the brain, it can be difficult to precisely manipulate *in vivo*. Modeling allows for precise control of internode lengths and distributions, facilitating investigation of a variety of myelination patterns. This approach is significant, as many models build on the underlying assumption that myelin patterns are uniform and homogeneous along axons.

The spatial arrangement of myelin sheaths is especially important when evaluating demyelination associated with aging and pathology, as well as potential remyelination following these insults. Reducing the length of a fraction of internodes to half their original length (representing remyelinated segments) has been used to assess the impact of segmental demyelination and remyelination (Scurfield and Latimer, 2018), given that remyelination following injury generates segments of shorter and thinner internodes (Lasiene et al., 2008, 2009). A double-cable model built by the same authors as the model used in Arancibia-Càrcamo et al. (2017) was employed, but instead fit to mammalian motor neurons (McIntyre et al., 2002). As expected, conduction velocity decreases as the fraction of remyelinated segments increases because remyelinated internodes are shorter than healthy ones. However, the change in conduction velocity was also shown to depend on the spatial arrangement of shorter and longer internodes. Indeed, assuming the same number of remyelinated segments, transitions between short and long internodes slow conduction velocity more than if an axon contained all of the shorter internodes on one half of the axon and all the longer internodes on the other. These findings were confirmed using the Gow and Devaux tight junction model, which explicitly includes tight junctions (Gow and Devaux, 2008). Double-cable models such as McIntyre et al. (2002) do not include tight junction dynamics, limiting their accuracy in modeling action potential conduction in axons with a diameter less than 0.9 μm (most myelinated fibers in the human brain are thinner than 1 μm; Liewald et al., 2014). To facilitate comparison between the results, a geometrically similar axon including properties such as node-to-node distance relative to axon diameter, g-ratio, and simulation environment was modeled to limit confounding variables.

More recent results further support the importance of myelin arrangement along the axon, further demonstrating that spatial heterogeneity of myelin sheathing impacts several properties of action potential conduction including conduction velocity and susceptibility susceptibility to propagation failure, as well as susceptibility of the axon to demyelination (Talidou and Lefebvre, 2025). The model is adapted from cable theory and uses an exponential integrate-and-fire model to simulate spike generation and conduction. Each axon has a given length and is covered by myelin sheaths of varying lengths such that the sum of myelinated compartments equals a predetermined myelin coverage percentage. Two classes of model axons were constructed, the first reflecting cortical myelination motifs and the second reflecting callosal myelination motifs. Given that myelin sheath length and organization vary greatly between brain regions, notably between the cortex and white matter circuitry (Glasser and van Essen, 2011; Thornton et al., 2024), the model incorporates mouse *in vitro* and *in vivo* imaging data to generate physiologically realistic sheath lengths and spatial arrangements (Chong et al., 2012). Other axonal and myelin parameters were fixed in order to isolate the effects of variability in myelin length and arrangement.

Axons with more uniform myelin sheath lengths and arrangements, such as those observed in the cortex, demonstrate negligible variability in conduction delays, whereas heterogeneous myelin motifs demonstrate high variability in conduction delays. This relationship between conduction delays and spatial heterogeneity is nonlinear and path-dependent, heavily depending on the arrangement and length of neighboring sheaths. Further exploration of this observation suggests that spatial heterogeneity increases the likelihood of action potential conduction failure, particularly when long unmyelinated segments are present. This makes some motifs more vulnerable to demyelination, which can result in neurological disorders (Duncan and Radcliff, 2016). In summary, the lengths and arrangements of myelin sheaths in the brain are highly variable, leading to differences in conduction velocity. This is consistent with previous findings on spatial heterogeneity of myelin sheaths in remyelinated axons (Scurfield and Latimer, 2018), and highlights how regulation of sheath organization is essential for coordinating spike timing required for healthy brain function.

Axonal ADM models, which are often biologically detailed, explicitly model action potential dynamics along individual myelinated axons composed of myelin sheath and node of Ranvier-like compartments, based on imaging observations in a variety of animal models (Ford et al., 2015; Arancibia-Càrcamo et al., 2017; Scurfield and Latimer, 2018; Talidou and Lefebvre, 2025). This class of models also uses the most biophysically detailed ion dynamics, often relying on Hodgkin-Huxley-like dynamics to simulate realistic ionic currents (Ford et al., 2015; Arancibia-Càrcamo et al., 2017; Scurfield and Latimer, 2018). Even in models using simplified phenomenological action potential conduction (Talidou and Lefebvre, 2025), the spike propagation dynamics along myelinated and unmyelinated segments agree with those of biophysically detailed models. The experimental constraints incorporated in the aforementioned axonal models make this body of work complementary to experimental studies, because they generate experimentally testable predictions about how the structural properties of myelin influence conduction timing. The major limitation of these models is that they do not explicitly model ADM. Instead, they treat myelin microstructure as a static parameter and model different myelination states as a proxy for the resulting outcome of ADM (and pathology).

However, while these models generally do not model ADM, they provide evidence for how plasticity in the structural properties of myelin can modulate conduction velocity and the timing of neural signaling. These results suggest that while each structural and architectural feature can increase or decrease conduction velocity, specific configurations can optimize spike timing, which is a primary goal of ADM. Recent experimental observations of mature myelin remodeling parallel this idea, as both node (Auer et al., 2018) and internode (Bacmeister et al., 2022) lengths have been shown to be dynamically regulated to support axonal propagation.

Axonal computational modeling isolates the various mechanisms by which ADM may dynamically remodel mature myelin, including how plasticity of node length, internode properties, and myelin coverage each contribute to altered spike timing. Experimental studies can then test these predictions and evaluate how axon-level changes in myelination influence downstream dynamical and behavioral phenomena in healthy and pathological contexts. Future axonal modeling work should aim to explicitly model ADM, ideally using a mechanistic learning rule that alters myelin structure based on neural activity. Modeling could also evaluate the effects of myelin remodeling and ADM in white matter tracts, which are known to play a significant role in brain function and behavior, but have distinct myelination patterns from gray matter regions.

### 2.2 Network models of ADM

#### 2.2.1 Microcircuit models

Despite their insights, single-cell models provide limited information on how ADM and neuron-glia coupling shape the dynamics of neural circuits. Emerging circuit-level datasets in gray matter reveal diverse dynamic phenomena accompanying myelin changes, ranging from hyperexcitability (Bacmeister et al., 2020) to altered firing rates and patterns (Madden et al., 2021; Dubey et al., 2022; Arafa et al., 2026). Network-level models are therefore better suited to investigating how ADM influences neural dynamics and function. These models often use conduction velocity as a proxy for myelination and explore how its modulation affects higher-level dynamics, such as synchrony, oscillatory activity, and information transmission between neurons or across different axons.

The relationship between ADM and spike timing has been directly examined by modeling the feedback loop between neural activity and myelination in a spiking neural network (Talidou et al., 2022). The model consists of a recurrent network of excitatory spiking neurons in which axonal conduction delays are state-dependent and modulated by activity-dependent changes in conduction velocity. While OL compartments were not explicitly modeled, neuron-OL feedback was still captured. A Hebbian, use-dependent plasticity rule was used to phenomenologically model the effect of firing rate on OL activity and its subsequent effects on conduction velocities. In this formulation, conduction velocity scales with firing rate along a given axon such that elevated firing increases myelin formation and conduction velocity, whereas reduced firing leads to myelin retraction and slower conduction.

Network simulations demonstrate how ADM acts as a homeostatic mechanism of gain control, through its ability to temporally coordinate action potentials and increase firing rates (Talidou et al., 2022). Indeed, ADM tunes conduction velocities to up-regulate correlated activity across spatial scales such that the temporal coordination of action potentials is optimized. To emulate the effects of myelin plasticity in response to stimuli as observed experimentally, the model was then extended to investigate how stimuli engage ADM to alter conduction velocities. ADM can bidirectionally and reversibly alter conduction velocities in response to external stimuli, suggesting that myelin plasticity in the brain responds dynamically to different states, such as learning versus rest (Talidou et al., 2022). Given that synaptic connectivity is fixed during simulation, the results highlight how timing plasticity can complement synaptic plasticity in maintaining optimal brain function and how ADM plays an important role in neural communication, a conclusion further supported by recent theoretical work demonstrating that adaptive myelination can stabilize neural dynamics (Lefebvre et al., 2025).

Microcircuit modeling has also been leveraged to determine how OL-mediated myelin plasticity modulates conduction velocity to optimize the timing and synchrony of action potentials propagating toward a postsynaptic target (Pajevic et al., 2023). Given that previous models of myelin plasticity are rate-based and do not account for individual spike timing (Talidou and Lefebvre, 2025), Pajevic et al. (2023) aimed to better understand how OLs integrate local spike timings as feedback to modulate conduction velocities. A model of OL-mediated myelin plasticity was built using measurements and observations from previous microscopy and imaging experiments demonstrating that each OL myelinates multiple axons. Although the physiological mechanisms of OLs are not explicitly modeled, this study is the first to phenomenologically model how OLs may respond to neural activity and subsequently tune conduction delays. In this framework, each OL releases a global intracellular signaling factor in response to an action potential propagating along any of the axons it myelinates. These action potential-triggered responses are temporally transient, allowing the model to integrate specific spike timing information for each action potential. When a spike occurs along one of the myelinated axons, a transient spike response signal is sent back to the OL. In response to multiple action potentials, the individual transients are integrated (termed the global signal), which provides a shared readout signal of spike activity and timing for each OL and all of the axons that it myelinates. To account for differences in myelination among axons myelinated by the same OL, each axon contains a unique local myelination factor. Release of the myelination factor occurs when an action potential occurs along the axon and is implemented such that the magnitude of release is proportional to the global signal. This allows selective, bidirectional modulation of myelination along each axon. The combination of global and local signals allows for the dynamic regulation of conduction velocities in response to action potentials, through feedback based on spike arrival timing at the level of OL processes.

Model simulations reveal that OLs can actively tune conduction velocities to selectively synchronize correlated spike trains (Pajevic et al., 2023). Meanwhile, the conduction velocity of uncorrelated spike trains along axons remains unaffected by this synchronization process. In addition, the synchronization of correlated signals in the model is greatest when the firing rate in axons is 10 Hz or less, and when the transient response of OLs to spikes is 10–40 ms (Pajevic et al., 2023). This suggests that there is an optimal regime of neural activity, namely low-frequency regimes, where state-dependent OL-mediated myelin plasticity tunes conduction velocities to support neural synchrony. Thus, computational modeling by Pajevic et al. (2023) proposes a biologically plausible mechanism of ADM in which OLs act as coincidence detectors of neural activity and subsequently tune conduction velocities through changes in myelination.

Microcircuit models are best suited to addressing how ADM reshapes circuit-level dynamics. They bridge the gap between highly biophysically detailed axonal models and primarily theoretical macroscopic brain models. They explicitly model how ADM translates neural activity, either through rate-based (Talidou and Lefebvre, 2025) or spike-timing-dependent myelination (Pajevic et al., 2023) learning rules, into changes in conduction velocity and spike timing. This approach has revealed that ADM can homeostatically and bi-directionally alter conduction velocity through changes in myelination, a mechanism by which spike timing can be correlated or decorrelated as action potentials reach their postsynaptic target. As demonstrated in both of these studies, this plays an important role in governing neural synchrony, a phenomenon essential to learning and communication in sensory systems requiring temporal precision (Talidou et al., 2022; Pajevic et al., 2023).

While these models are novel and greatly advance our understanding of ADM and myelin plasticity, there are many avenues for future work. As previously mentioned, this class of models often implements a phenomenological learning rule to approximate how OLs govern ADM. This is in part due to biological uncertainty surrounding the relationship between neural activity, OL activity, and myelin plasticity, but future modeling work should aim to incorporate these rich biological mechanisms where possible to build a more mechanistically complete picture of ADM. Further, given that OLs themselves impact neural activity (Dutta et al., 2018), it is highly likely they are involved in this homeostatic ADM process. Second, unlike axonal models, microcircuit models bypass explicitly modeling myelin sheaths and changes to their structure. Rather, they approximate changes in myelin structure through changes in conduction velocity. Modeling how ADM leads to changes in myelin structure and architecture would improve the connection between ADM and structural myelin plasticity. Thirdly, the existing class of microcircuit models is either a purely excitatory network (Talidou and Lefebvre, 2025) or a bundle of excitatory axons (Pajevic et al., 2023). This ignores the complex interactions within excitatory-inhibitory networks, which not only provide feedback to one another, but also contain differing levels of myelination (Zonouzi et al., 2019; Basu et al., 2023). Finally, ADM in biological systems spans timescales ranging from millisecond action potential timings to changed in myelin structure over hours to days. These timescales are compacted in modeling for computational tractability, but this leaves a gap in physiological relevance that can be difficult to address. However, recent advances in spiking neural networks with adaptive delays (Coombes et al., 2026) may circumvent some of these limitations and provide a tractable mathematical framework for exploring the relationship between ADM and neural synchrony. Ultimately, current limitations highlight the challenge of balancing biological realism with computational and mathematical tractability, emphasizing the need for combined experimental-computational methods to study ADM.

#### 2.2.2 Large-scale models

Most inter-regional neural traffic takes place through the white matter, a heavily myelinated network of nerve fibers that occupies roughly half of the mammalian brain volume and is responsible for relaying nerve impulses between brain regions. White matter scaffolding has an intrinsic capacity to reorganize through ADM (Bells et al., 2017, 2019). Unlike models operating at axonal and microcircuit scales, large-scale white matter ADM models (Pajevic et al., 2014; Noori et al., 2020; Park and Lefebvre, 2020) do not explicitly represent individual neurons and instead adopt a more phenomenological formulation through neural masses (Deco et al., 2008). This approach provides a more direct link to neuroimaging data such as functional magnetic resonance imaging (fMRI), electroencephalography (EEG), and magnetoencephalography (MEG). It is further motivated by observations that white matter structure and integrity are both critical in supporting synchronous dynamics and oscillatory coordination across brain areas (Fields, 2015; Bells et al., 2017, 2019). In their seminal review, Pajevic et al. (2014) emphasize that time delays, which vary as a consequence of myelin-dependent changes in conduction velocity, can strongly influence brain oscillations and synchrony. They support this view by drawing on a phenomenon demonstrated in a variety of phenomenological coupled oscillator models, whereby dispersed time delays suppress synchrony and oscillatory activity. This phenomenon, referred to as *amplitude death* (Atay, 2003; Coombes and Laing, 2009; Kyrychko et al., 2012; Wang and Campbell, 2018), links dispersion in time delays to the suppression of oscillations and stabilization of non-oscillatory network states.

Time-delayed Kuramoto-type oscillator networks provide a well-known example of amplitude death. The Kuramoto model is a macroscopic mathematical model consisting of a large network of coupled oscillators (Kuramoto, 1984; Strogatz, 2000), and is routinely used as a neural mass model to study synchronization (Atay, 2003; Breakspear et al., 2010; Ponce-Alvarez et al., 2015; Petkoski et al., 2018). Each oscillator can be interpreted as a population of neurons or a brain region and operates at a given natural frequency. Oscillators that are weakly coupled are primarily asynchronous, although increasing coupling generally causes the transition to a synchronous state.

Using the Kuramoto model with a single, constant delay, small changes in myelin can profoundly alter neural oscillations: for example, millisecond-scale changes in time delays can impede the ability of neural assemblies to synchronize (Pajevic et al., 2014), reinforcing the notion that myelin-dependent delays influence large-scale synchrony and oscillatory coordination across the white matter (Deco et al., 2009; Cabral et al., 2011, 2014; Bells et al., 2017, 2019; Sayli and Coombes, 2024). Consistent with theoretical predictions (Yeung and Strogatz, 1999; Breakspear et al., 2010; Park and Lefebvre, 2020), their simulations also reveal that conduction delays directly alter oscillation frequency. These findings suggest that in addition to adaptive synaptic strengths, myelin plasticity in the brain may tune conduction velocity to regulate oscillatory activity between brain areas (Fields, 2015). Although ADM is not explicitly modeled in this version of the Kuramoto network, dysregulation of time delays induced by changes in myelination can significantly alter the brain's dynamical regime and may play a role in neurological disorders involving synchrony, such as epilepsy and schizophrenia (Coombes and Laing, 2009; Takahashi et al., 2011; Campbell and Kobelevskiy, 2012; Kömek et al., 2012; Kirli et al., 2014; Bells et al., 2017, 2019; Liu et al., 2019), suggesting a potential homeostatic role of ADM in neural systems.

Noori et al. take a step further by investigating how ADM regulates oscillatory neural activity to maximize neural communication efficiency (Noori et al., 2020). The Kuramoto model is again used for its ability to model neural oscillators and study synchronization, but this time with spatially distributed and state-dependent delays (as opposed to a single constant delay). This model was further informed by macaque white matter structural connectivity, where each oscillatory unit represents one of the 80 cortical or 16 subcortical regions of the human brain. To describe the effects of ADM and neuron-OL coupling, a phenomenological learning rule was implemented in which conduction velocity along each axon changes according to the oscillatory phase difference between nodes, thereby implementing a slow feedback loop that links phase coherence and conduction velocity. While this model does not account for individual neural dynamics or the underlying biological mechanisms responsible for generating oscillations or myelin modification by OLs, it provides insight into the macroscopic relationship between ADM and network-level synchrony.

Simulations reveal how the inclusion of ADM enables network synchronization, allowing an initially incoherent network to self-regulate conduction velocities and achieve a globally phase-locked, coherent state (Noori et al., 2020). Furthermore, variability in conduction velocity compensates for differences in axonal tract lengths to maintain statistically similar conduction delays needed for synchronization: a homeostatic mechanism steering the network away from amplitude death. Noori et al. (2020) also show that adaptive conduction velocities stabilize phase-locked states across a wide range of conditions. Synchronization requires high synaptic strengths and is limited to low frequency bands in the absence of myelin plasticity, whereas introducing myelin plasticity enables synchronization at both lower synaptic strengths and across wider frequency bands (Noori et al., 2020). Further, in response to network perturbations, insults, or injury induced by selectively removing network connections, plastic conduction velocities can self-adjust to restore the phase-locked synchrony that was destabilized by the perturbation (Noori et al., 2020). In summary, adjustment of conduction velocity through ADM may provide a glial mechanism by which large-scale brain networks can self-organize to maintain homeostasis and resilience. While many of the rich biological processes in ADM were bypassed in this neural oscillator model, it nonetheless revealed the net effect of ADM (using plastic conduction velocities) on brain-scale dynamics, opening the door to future, more biologically detailed ADM models (Talidou et al., 2022; Pajevic et al., 2023).

Lastly, Park and Lefebvre revisited the work of (Noori et al., 2020) by performing a theoretical analysis on the Kuramoto model with plastic, phase-dependent delays, specifically characterizing the stability of globally synchronous states (Park and Lefebvre, 2020). To address this challenging mathematical problem, they developed a stability criterion for globally synchronous solutions and its dependence on the plasticity of time delays in two and higher dimensions. These analyses confirm that phase-dependent delays increase the stability of globally synchronous states, further promoting the resilience of oscillatory communication to perturbations such as the loss of axonal connections. More recent theoretical work using pulse-coupled oscillators demonstrated that delays can lead to the occurrence of slow oscillations, which are impossible to obtain without adaptive delays (Klinshov and Nekorkin, 2024).

Large-scale neural oscillator models present some of the earliest computational investigations into ADM (Pajevic et al., 2014; Noori et al., 2020). These models reveal that ADM is a crucial mechanism organizing delays in the brain, laying the groundwork for future computational and experimental investigation. However, of the three listed classes of ADM models, large-scale models represent the most abstract formulation. At present, they often build on statistical physics frameworks and/or principles, resulting in lower biological realism compared to other classes of models. For example, oscillatory units are used to model different brain regions (Pajevic et al., 2014; Noori et al., 2020). That is, they do not explicitly model individual cells and their activity, instead approximating neural activity using oscillator phase as the state variable. Similar to microcircuit models, studies incorporating ADM use phenomenological learning rules that ignore the complex biology underlying ADM (Noori et al., 2020; Park and Lefebvre, 2020). Finally, these models similarly compress the time required for changes in myelination compared to what is observed *in vivo*.

Despite these limitations, large-scale models highlight the significance of ADM and glial cells in supporting oscillatory activity in large-brain networks. Taken together, these computational and mathematical studies similarly suggest that ADM-mediated adaptive conduction delays in the white matter are critical for regulating large-scale synchrony and increasing resilience to perturbations or injury, aligning with experimental work suggesting that white matter integrity is essential for brain function and that its disruption can lead to cognitive deficits (Knowles et al., 2022a,b).

## 3 Toward a unified framework of activity-dependent myelination

ADM has emerged as a central focus of contemporary neuroscience, fundamentally reshaping how neural computation and neurological disorders are conceptualized (Fields, 2015; Hughes et al., 2018; Mitew et al., 2018; Bacmeister et al., 2020; Knowles et al., 2022a,b). By enabling myelin to dynamically tune action potential conduction, ADM introduces a powerful mechanism of neuroplasticity operating across both gray matter and distributed white matter networks, extending plasticity beyond the synapse. As summarized in Table 1, existing ADM models span multiple levels of biological realism and spatial scales, reflecting diverse functional objectives and underlying mechanisms. While these models have generated critical insights into ADM and myelin plasticity more broadly, the field remains fragmented, underscoring the need for a more unified and integrative computational framework. Next, we highlight some of the outstanding challenges of ADM modeling, and directions that we believe can inform future experimental analysis. This iterative, interdisciplinary approach can then refine models, strengthen the link between experiments and modeling, and accelerate the development of a unified mechanistic understanding of ADM.

**Table 1:** Summary of ADM models.

	Model	Detail	ADM Mechanism	Findings
Axonal	Ford et al. (2015)	Biologically detailed	None	Internode length adjustment tunes axonal conduction velocities
	Arancibia-Càrcamo et al. (2017)	Biologically detailed	None	Node of Ranvier length adjustment tunes conduction velocities more efficiently than internode length
	Scurfield and Latimer (2018)	Biologically detailed	None	Distribution of short and long internodes influences conduction velocity
	Talidou and Lefebvre (2025)	Biologically detailed	None	Spatial variability of myelination is essential to support precise conduction timing
Microcircuit	Talidou et al. (2022)	Mixed	Conduction velocity scales proportionally to firing rate	ADM bidirectionally alters conduction velocities in response to external stimuli
	Pajevic et al. (2023)	Mixed	Global myelination signaling factor and local myelination factor	OLs actively tune conduction velocities in response to neural activity
Macroscopic network	Pajevic et al. (2014)	Phenomeno logical	None	Myelin plasticity tunes conduction velocities to modulate oscillatory activity
	Noori et al. (2020)	Phenomeno logical	Phase-dependent conduction velocity adjustment	ADM tunes conduction velocities to homeostatically regulate synchrony
	Park and Lefebvre (2020)	Phenomeno logical	Phase-dependent conduction velocity adjustment	Phase-dependent delays stabilize synchronous activity and promote resilience to perturbations

### 3.1 Incorporating biological richness in neural networks

Most notably, the physiological mechanisms underlying ADM remain unclear, from both experimental and computational perspectives. This is perhaps unsurprising, given that ADM links neural activity and myelin formation, complex biological processes that involve the interaction of many signaling pathways, and cell types. For instance, current ADM research primarily focuses on the interaction between OLs and neurons (Talidou et al., 2022; Pajevic et al., 2023). However, OLs and their activity have yet to be modeled explicitly, with existing models instead relying on *ad hoc* and/or phenomenological learning rules, which limits their ability to establish candidate mechanisms underlying ADM. As such, future integration of OL activity into ADM models will depend heavily on experimental progress. Current ADM models change myelination based on firing rate (Talidou et al., 2022), individual spike timings (Pajevic et al., 2023), or synchrony (Noori et al., 2020). Experimental measurements quantifying OL responses to neural activity, including sensitivity to different firing frequencies, patterns, and durations, combined with imaging studies that analyze the pathways OLs use to convert activity into changes in myelination, can help refine the learning rules linking activity and myelination. Future ADM models should aim to better capture biological OL-axon signaling in their plasticity rules. Another OL-related area of interest is OL heterogeneity, which is presently ignored in ADM models. Existing models assume all axons respond to neural activity and change myelination identically, unlike what is observed experimentally (Mitew et al., 2018; Marisca et al., 2020). Future models should assess the network effects of heterogeneous myelination, which has already been shown to impact conduction dynamics along individual axons (Scurfield and Latimer, 2018; Talidou and Lefebvre, 2025).

Despite the focus on OLs in ADM, recent research also implicates other glial cells, including astrocytes, OPCs, and microglia, in modulating neural activity and myelination (Dutta et al., 2018; Pan et al., 2024; Kedia et al., 2024). The contribution of these other glial cells is currently neglected in modeling studies, representing an avenue of exploration that may benefit future work. Additionally, current ADM modeling studies often generalize findings across brain regions. However, myelination patterns are known to vary widely across cell types and brain regions (Tomassy et al., 2014; Talidou and Lefebvre, 2025). These differences suggest that myelin plasticity may obey fundamentally distinct optimization rules across different brain regions and white matter pathways.

Lastly, a topic of recent interest in the myelin plasticity space yet to be characterized in ADM models is calcium-dependent signaling. Conditional deletion of L-type calcium channels in mouse OPCs significantly reduces postnatal myelination and remyelination ability in adults, initially suggesting a link between calcium and myelination (Cheli et al., 2016; González et al., 2017). Recent *in vitro* recordings and imaging suggest that OPCs integrate and respond to synaptic input by increasing their intracellular calcium concentration, a cue that may mediate neuron-glia coupling and thereby guide ADM (Sun et al., 2016). Recent *in vivo* imaging builds on this finding and demonstrates that myelin sheath plasticity depends on intracellular calcium concentration, which in OLs is influenced by neuronal activity (Krasnow et al., 2018; Baraban et al., 2018). Specifically, increases in intracellular calcium transients, defined as brief periods of increased local calcium concentration, within developing OLs trigger myelin sheath elongation, while sheath shortening is associated with a lower frequency of calcium transients (Krasnow et al., 2018). Taken together, although further investigation is needed, calcium-driven signaling may provide an underlying mechanistic link for ADM, whereby a calcium-mediated feedback loop between neuronal activity and myelination regulates myelin formation and optimization. If confirmed, it would establish calcium signaling as a mechanism that temporally bridges fast neural activity and slower changes in myelination.

### 3.2 Bridging scales with multi-scale modeling

As mentioned previously, ADM operates across a wide range of temporal (milliseconds to days) and spatial (microns to meters) scales, all within circuits of immense complexity. Understanding the mechanisms and consequences of myelin remodeling on brain dynamics and function, and vice versa, thus requires a multiscale approach, with modeling as a central tool.

Changes in myelination occur over hours and days in animal models, whereas neural activity can occur in milliseconds. This timescale mismatch presents an experimental challenge since long-term *in vivo* myelin imaging in parallel with continuous electrophysiological recordings is often expensive or infeasible. As such, establishing a mechanistic link is difficult, but modeling can help bridge this gap. Existing ADM models attempt to bridge these timescales (Noori et al., 2020; Talidou et al., 2022; Pajevic et al., 2023), but compress the timescale of changes in myelination. This highlights a major challenge that remains: systems with state-dependent time delays, such as myelinated neural circuits, are among the most difficult to characterize mathematically. Our limited understanding of these systems significantly hampers data interpretation. Rather than trying to precisely model every temporal detail of ADM, modeling should instead focus on preserving the relative qualitative dynamics and biophysical relationships underlying ADM. This again highlights the strength of interdisciplinary methods, in which modeling provides testable mechanistic predictions to guide experiments, while experiments provide the biological constraints that ensure any model simplifications still preserve biological realism.

The spatial multiscale nature of ADM presents an analogous challenge in developing a unified theory of the mechanism. ADM-related processes range from changes in molecular signaling at the OL and axon levels, to gray and white matter circuits that link brain regions. Experimental techniques are typically best suited to investigating one of these spatial scales. Improved imaging techniques, including two-photon microscopy, and novel techniques such as neuropixels recordings have helped bridge the gap between cellular- and circuit-level scales. However, additionally linking these to cellular-level measurements such as calcium imaging, and whole-brain measurements obtained through MRI, fMRI, and EEG remains a challenge. Computational modeling can help bridge the gap between spatial scales using a single framework. Indeed, recent neural modeling by Ibañez et al. begins to address this connection, even extending to work on myelin alterations (Ibañez et al., 2024). A multi-compartmental axonal model with myelinated and unmyelinated segments, similar to those covered in the axonal models section above, was first constructed using experimental measurements from dorsolateral prefrontal cortex (dlPFC) layer 3 pyramidal neurons in the rhesus monkey. Action potential generation and propagation were governed by Hodgkin-Huxley-style ion channel kinetics. To evaluate network scale influence such as effects on working memory, they then constructed a spiking E-I network of leaky integrate-and-fire neurons. The network model was similarly fit to rhesus monkey dlPFC data, to bridge the gap between spatial scales when studying myelin alterations and cognition.

Although not an explicit ADM model, this work is relevant in the context of ADM as it demonstrates that, at the axonal level, demyelination leads to slower conduction velocity and introduces propagation failure (Ibañez et al., 2024). In tandem, at the network level, propagation failures caused by demyelination were shown to impair working memory, but can be restored with remyelination (Ibañez et al., 2024). This illustrates how changes in myelin structure can affect axonal conduction, and the subsequent impact on circuit-level dynamics and behavior. Future ADM modeling should adopt multiscale frameworks that can link biological processes occurring across different spatial scales. Quantitative observations and hypotheses on mechanistic ADM pathways generated from modeling can then be tested experimentally to validate or refute how ADM translates to whole-brain dynamics and behavior.

## 4 Conclusion

Myelin is a dynamic structure that plays an essential role in modulating the temporal precision of neural signaling. ADM is an emerging area of research that challenges the traditional view that myelin is static by demonstrating its ability to modify conduction velocity, spike timing, and synchrony in response to neural activity. Addressing outstanding gaps in knowledge to create a unified framework for ADM will require interdisciplinary approaches, given the challenge of bridging spatial and temporal scales. While the mechanisms underlying ADM remain unclear, we believe that calcium dynamics may provide the link between neural activity and OL activity in remodeling myelin structure. Given the dynamic nature of ADM, future models should include dynamic conduction delays, analogous to widespread adoption of variable synaptic weights, to explore how myelin plasticity interacts with neural dynamics. Just like synaptic plasticity models, ADM models may help clarify the role of this form of plasticity in healthy and pathological brain states, while inspiring new applications in engineering, machine learning, and AI.

## Data Availability

The original contributions presented in the study are included in the article/supplementary material, further inquiries can be directed to the corresponding authors.
